# Cellular senescence promotes cancer metastasis by enhancing soluble E-cadherin production

**DOI:** 10.1016/j.isci.2021.103022

**Published:** 2021-08-24

**Authors:** Koichiro Kawaguchi, Kaori Komoda, Ryuta Mikawa, Azusa Asai, Masataka Sugimoto

**Affiliations:** 1 Research Institute, National Center for Geriatrics and Gerontology, Aichi 474-8511, Japan; 2 Nagoya University Graduate School of Medicine, Nagoya 466-8550, Japan

**Keywords:** cell biology, cancer

## Abstract

Cellular senescence acts as a potent tumor-suppression mechanism in mammals; however, it also promotes tumor progression in a non-cell-autonomous manner. We provided insights into the mechanism underlying senescence-dependent metastatic cancer development. The elimination of senescent cells suppressed the lung metastasis of melanoma cells. Using an antibody array screening of humoral factor(s) that depend on cellular senescence, we identified soluble E-cadherin (seCad) as a potential mediator of the senescence-induced melanoma metastasis. seCad enhanced the invasive activity of melanoma cells both *in vitro* and *in vivo*, and gene expression profiling revealed that seCad induced genes associated with poor prognosis in patients with melanoma. An analysis of sera from patients revealed that serum seCad is associated with distant metastasis. Our data suggest that senescent cells promote metastatic lung cancer through seCad, and that seCad may be a potential diagnostic marker as well as a therapeutic target for metastatic lung cancer.

## Highlights

• Elimination of senescent cells diminished lung metastasis of B16-F10 melanoma cells
• Senescent cells enhanced ectodomain shedding of the E-cadherin protein
• The resultant soluble E-cadherin (seCad) promotes lung metastasis
• Plasma seCad level is associated with metastasis in patients with melanoma

## Graphical abstract

**Figure:**
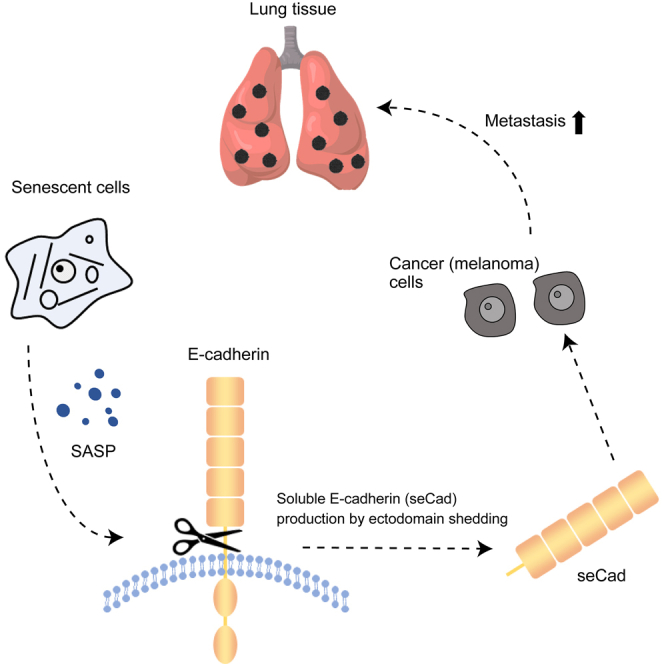

## Introduction

Senescent cells accumulate in tissues during aging and are thought to underlie tissue aging (Dimri et al., 1995; Krishnamurthy et al., 2004). Cellular senescence is involved in the onset of several aging-associated diseases (Munoz-Espin and Serrano, 2014), and the senescence-associated secretory phenotype (SASP) plays a key role in the establishment of pathologies (He and Sharpless, 2017). Recent studies that used semi-genetic or pharmacological ablation of senescent cells have provided further insights into the pathophysiological roles of cellular senescence in aging-associated disorders. The elimination of senescent cells extended the health span by alleviating the aging-associated phenotypes (Baar et al., 2017; Baker et al., 2016; Farr et al., 2017; Xu et al., 2018) and pathologies in disease models (Childs et al., 2016; Jeon et al., 2017; Ogrodnik et al., 2017; Roos et al., 2016; Schafer et al., 2017). We have also established a transgenic mouse line, ARF-DTR, that expresses a diphtheria toxin (DT) receptor and luciferase under the control of the *Arf* promoter/enhancer. Using ARF-DTR mice, we discovered that the elimination of p19^Arf^-expressing cells from lung tissues restored pulmonary function in old animals (Hashimoto et al., 2016) and protected against elastase or cigarette-smoke-induced emphysema (Mikawa et al., 2018, 2020).

Although there is no doubt that senescence acts as a potent cell-autonomous tumor-suppression mechanism in mammals (Ben-Porath and Weinberg, 2005), it has become evident that senescent cells are able to promote the growth and invasion of neighboring cancer cells in a non-cell-autonomous fashion, at least partly through SASP (Coppe et al., 2008; Krtolica et al., 2001). Thus, senescence also contributes to malignancy by enhancing the metastasis of cancer cells. Metastasis to distal sites, including the lung, is a common feature of malignant melanoma, which accounts for the majority of the deaths among these patients (Bedrosian et al., 2000). Although the landscape of genetic alterations and driver mutations has been characterized in melanoma (Cancer Genome Atlas, 2015; Hodis et al., 2012), the mechanisms underlying the metastasis of melanoma are less understood. Increased age leads to a poor prognosis in patients with melanoma (Tsai et al., 2010); however, the involvement of cellular senescence in the metastasis of melanoma remains unknown.

E-cadherin is a type 1 transmembrane protein with a molecular weight of 120 kDa that plays a pivotal role in the dynamics of intercellular adhesion of epithelial cells. Deregulation of cadherin is a hallmark of cancer, as cadherin-mediated cell adhesion is involved in the diverse cellular processes that are associated with cancer progression, including cell proliferation, migration, and invasion (Jeanes et al., 2008). Ectodomain shedding of E-cadherin by extracellular proteinases, such as the A Disintegrin and Metalloproteinase (ADAM) and Matrix Metalloproteinase (MMP) families of proteinases (David and Rajasekaran, 2012), results in the production of the 80 kDa soluble E-cadherin (seCad). An increased level of seCad is observed in patients with cancer, including melanoma, which predicts that seCad may be used as a potential biomarker of malignancies (De Wever et al., 2007). The biological function of seCad is not well understood; however, recent studies have reported potential roles of seCad in cancer progression. seCad has been shown to interact with and activate the receptor tyrosine kinases, including the epidermal growth factor receptor (EGFR) and the insulin-like growth factor receptor (IGFR) (Brouxhon et al., 2014b; Inge et al., 2011). Furthermore, seCad has the ability to induce angiogenesis by activating β-catenin and NF-κB signaling in vascular endothelial cells, thereby promoting tumor proliferation and metastasis (Tang et al., 2018).

Here, we showed that seCad mediates the senescence-induced metastasis of melanoma. We investigated the linkage between the non-cell-autonomous effects of cellular senescence and cancer metastasis using a mouse melanoma metastasis model. The elimination of p19^Arf^-expressing senescent cells from lung tissues significantly reduced the lung metastasis of melanoma cells. We found that the presence of p19^Arf^-expressing cells promoted seCad production. Moreover, seCad enhanced the invasive activity of melanoma cells both *in vitro* and *in vivo*, whereas its inhibition suppressed their metastasis. The analysis of human sera identified a correlation between serum seCad level and melanoma metastasis. Thus, these results suggest that cellular senescence promotes the ectodomain shedding of E-cadherin; the resultant seCad contributes to senescence-induced cancer progression, and that seCad is a potential therapeutic/preventive target for malignant melanoma, as well as a biomarker for prognosis prediction.

## Results

### Aging-associated p19^Arf^-expressing cells promote the lung metastasis of B16-F10 melanoma cells in mice

**Figure 1:**
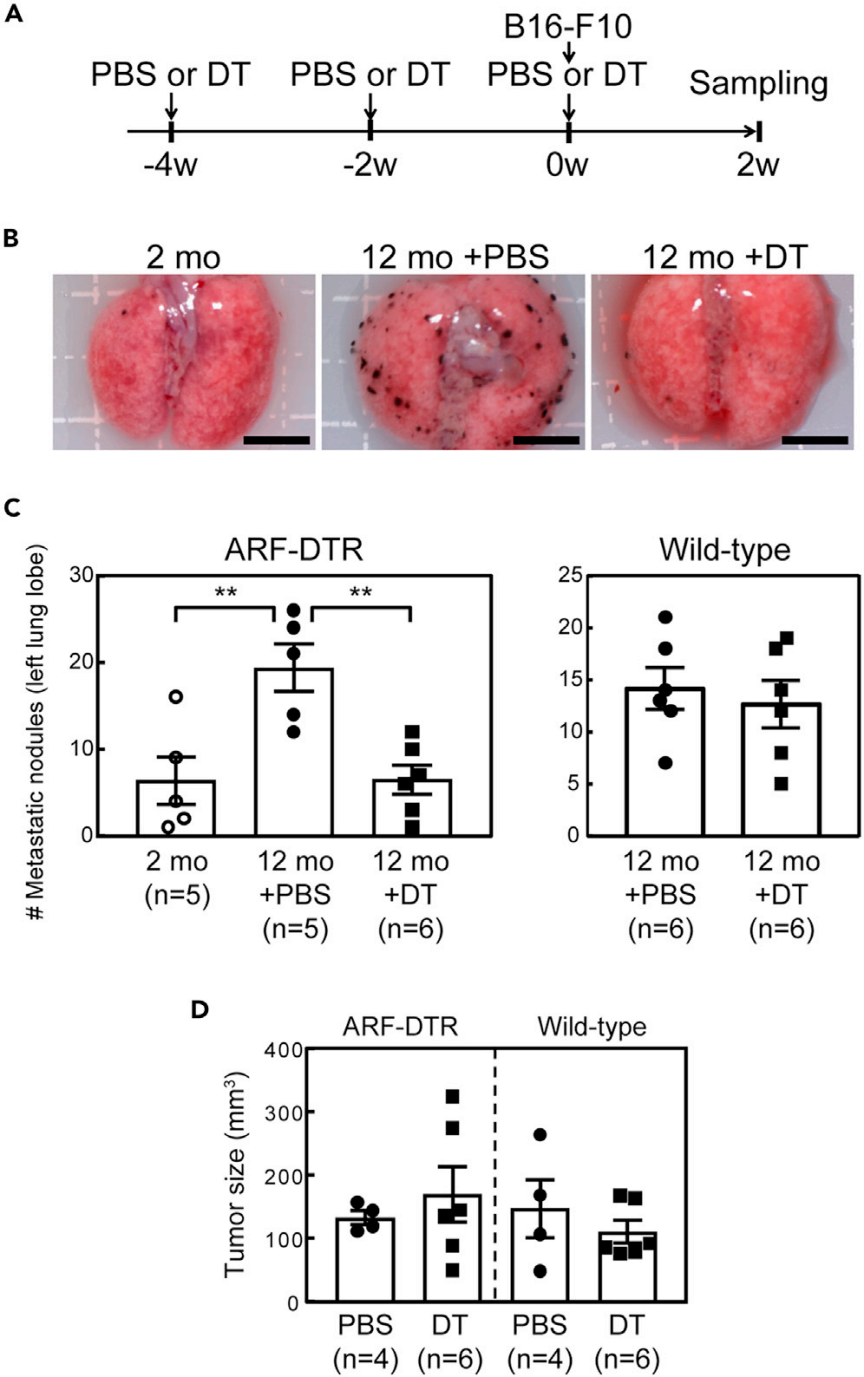
The elimination of p19^Arf^-positive cells suppressed the metastasis of melanoma cells to the lung (A) Experimental schedule. Twelve-month-old ARF-DTR or wild-type mice were administered DT or PBS intraperitoneally. B16-F10 mouse melanoma cells were injected into the tail vein. (B) Representative images of lung metastases of B16-F10 cells. Lung tissues were resected 2 weeks after the melanoma injection. Scale bar, 5 mm. (C) Number of lung metastatic nodules. The left lung lobe of each mouse was dissected under a stereoscopic microscope and the number of metastatic tumor nodules was counted. (D) Subcutaneous tumor sizes. B16-F10 cells were subcutaneously injected into 12-month-old ARF-DTR or wild-type mice. The tumor size was measured 2 weeks after transplantation. Data represent the mean value ±SEM in each group. Data were analyzed by one-way ANOVA and Tukey post-hoc analysis. Student's *t*-test was performed for the comparison of two groups. ∗∗p < 0.01. See also Figure S1.

To investigate the effects of p19^Arf^-expressing cells on lung metastasis, B16-F10 mouse melanoma cells were injected into the tail vein of wild-type or ARF-DTR mice (Figure 1A), and the number of tumor nodules in lung tissues were counted. The lung metastasis of the B16-F10 cells was significantly suppressed in DT-treated ARF-DTR mice, but not inwild-type mice (Figures 1B and 1C). These results were similar to those of a previous report by Demaria et al., which indicated that ablation of p16^Ink4a^-expressing senescent cells suppressed lung tumor metastasis in p16-3MR mice (Demaria et al., 2017). We have previously shown that DT treatment leads to the downregulation of *Ink4a* and *Arf* in old ARF-DTR mice, suggesting that the elimination of p19^Arf^-expressing cells results in the ablation of p16^Ink4a^-expressing senescent cells (Hashimoto et al., 2016; Mikawa et al., 2018, 2020). To confirm that *Arf* is associated with cellular senescence in mouse tissues, we analyzed the localization of p19^Arf^ and p16^Ink4a^ in the ARF-DTR lung. p19^Arf^ was observed in cells expressing p16^Ink4a^ in old lung tissues, and neither of these proteins was detected in lung tissues of young animals or DT-treated old animals (Figure S1A). Senescent-associated β-galactosidase (SA β-gal) was also detected in the old ARF-DTR lungs, but was absent or expressed at very low level in young or DT-treated tissues, respectively (Figures S1B and S1C). In addition, our previous report also revealed that p19^Arf^ is colocalized with γH2AX in old lung tissues (Hashimoto et al., 2016). Taken together, these results suggest that p19^Arf^-expressing cells represent cellular senescence in mice, and that their accumulation enhances the lung metastasis of the B16-F10 cells *in vivo*.

During aging,the expression of *Arf* and *Ink4a* increases in the lung and other tissues (Hashimoto et al., 2016; Krishnamurthy et al., 2004). Here, B16-F10 cells showed lower metastatic activity in young animals (Figures 1B and 1C), further supporting the notion that p19^Arf^-expressing cells promote lung metastasis. In contrast, the presence of p19^Arf^-expressing cells did not appear to promote the proliferation of cancer cells *in vivo*, as the size of the tumor resulting from subcutaneously transplanted B16-F10 cells or the size of metastatic nodules were not affected by DT treatment in ARF-DTR or wild-type mice (Figure 1D and data not shown).

We also examined the possibility that lung metastasis of B16-F10 cells might affect the p19^Arf^-expressing cells in lung. *In vivo* luciferase imaging analysis of ARF-DTR mice revealed that the lung luciferase activity, which represents the p19^Arf^-expressing cells in these mice (Hashimoto et al., 2016), were unchanged by the B16-F10 cell injection, suggesting that lung metastasis does not immediately affect the dynamics of cellular senescence (Figure S1D).

### p19^Arf^-dependent production of soluble E-cadherin in aged animals

**Figure 2:**
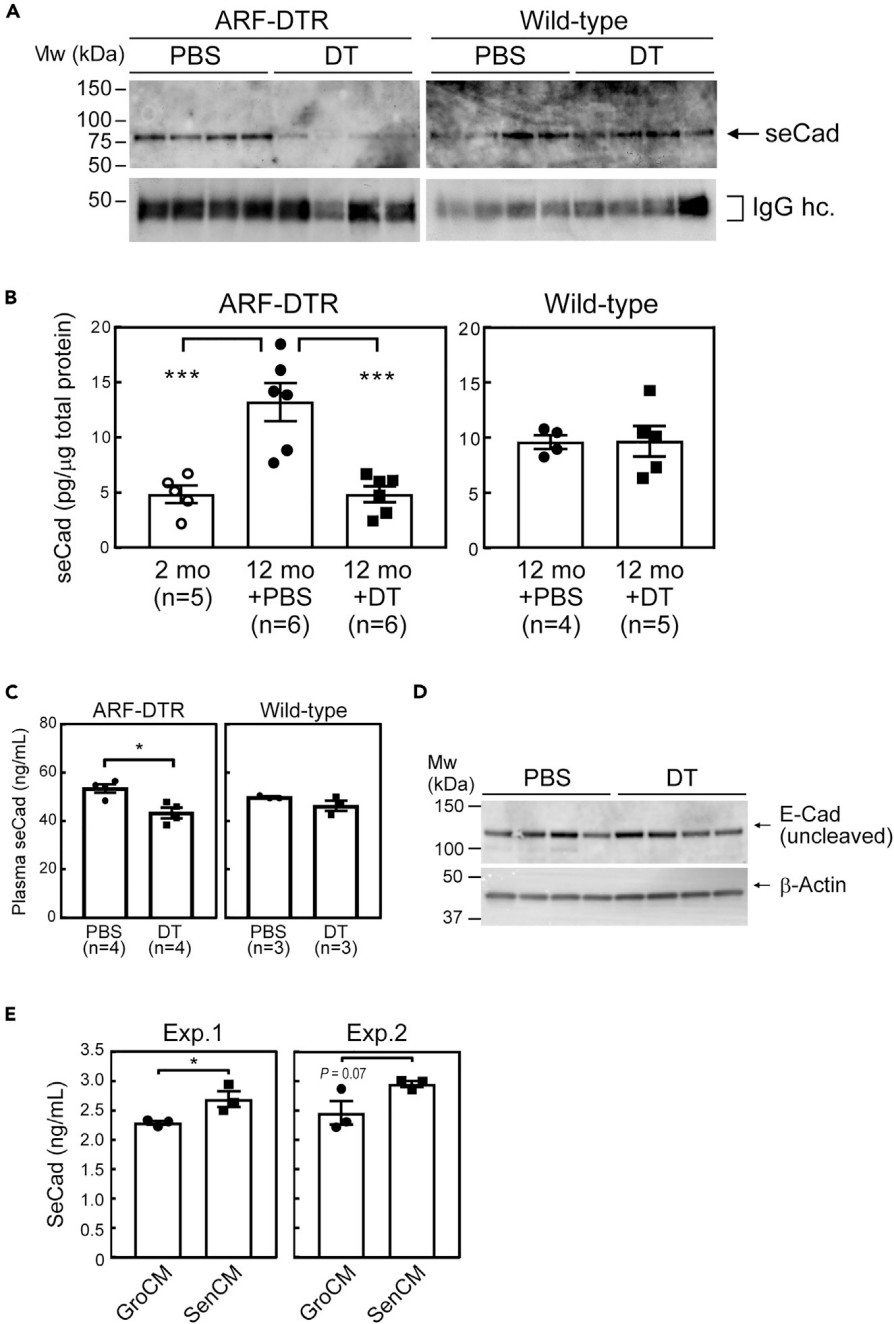
seCad levels were associated with p19^Arf^-expressing senescent cells (A) The seCad levels in the BALF of ARF-DTR or wild-type mice treated with DT or PBS were analyzed by immunoblotting. Four mice in each group were analyzed. IgG heavy chain (hc) was used as a loading control. (B and C) The seCad levels in the BALF (B) and plasma (C) were analyzed by ELISA. BALF seCad levels were normalized to protein content in each sample. (D) Lysates prepared from ARF-DTR lung tissues were analyzed regarding the uncleaved E-Cad level by immunoblotting. Four mice in each group were analyzed. β-Actin was used as a loading control. Data are presented as the mean values ±SEM in each group. (E) A549 cells were cultured in conditioned medium of non-senescent MEFs (GroCM) or senescent MEFs (SenCM) for 48 hr. The seCad levels in medium were analyzed by ELISA for human E-cadherin. The results of two independent experiments are shown. Data are presented as the mean values ±SD in each group. Data were analyzed by one-way ANOVA and Tukey post-hoc analysis. Student's *t*-test was used for the comparison of two groups. ∗p < 0.05 and ∗∗∗p < 0.001. See also Figures S2 and S3.

Although senescent cells accumulate in tissues during aging, their population is low even in old animals or human tissues (Dimri et al., 1995; Krishnamurthy et al., 2004). p19^Arf^ expression was observed in 1%–2% of mesenchymal cells in old mouse lung tissues, and it was also induced in epithelial cells when mice were challenged by cigarette smoke (Hashimoto et al., 2016; Mikawa et al., 2020). Therefore, it is plausible that the promotion of lung metastasis is exerted through the non-cell-autonomous effects of p19^Arf^-expressing cells. In fact, senescent cells have been shown to possess the ability to promote tumor growth through SASP (Ohtani, 2019). Therefore, we performed an antibody array screen to identify humoral factor(s) that are enhanced or suppressed by DT in the bronchoalveolar lavage fluid (BALF) of ARF-DTR mice. Among the 144 factors on the array, E-cadherin exhibited the most significant association with the p19^Arf^-expressing cells (Figure S2). E-cadherin normally exists as a membrane-bound protein, and its ectodomain shedding to produce seCad is mediated by extracellular proteinases, such as ADAM and Mmp (David and Rajasekaran, 2012). To validate the assumption that the signals detected in the antibody array reflected the changes in seCad, the BALF was analyzed by immunoblotting. Although the uncleaved membrane-associated E-cadherin (molecular weight, ∼120 kDa) was not observed in the BALF, seCad (∼80 kDa) was detected in this sample (Figure 2A). The administration of DT resulted in the downregulation of the 80 kDa seCad in the BALF of ARF-DTR mice, whereas no change was observed in wild-type samples. An enzyme-linked immunosorbent assay (ELISA) further confirmed these results; however, seCad was maintained at lower levels in the BALF of young animals (Figure 2B). Similarly, the plasma seCad levels were also downregulated after DT treatment in adult ARF-DTR, but not in wild-type animals, although the magnitude of the response was smaller than that of the BALF (Figure 2C).

Next, we examined whether the changes in seCad levels were attributable to the E-cadherin levels. Immunoblotting of lung total lysates revealed no detectable changes in the levels of membrane-bound E-cadherin between control (PBS) and DT-treated samples, suggesting that the increase in seCad level was caused by the increase in ectodomain shedding, rather than the increase in total E-cadherin (Figure 2D). In support of this notion, the *Adam10* mRNA, which encodes a major ectodomain-shedding proteinase, was downregulated in lung tissues after the elimination of p19^Arf^-expressing cells (Figure S3).

To further investigate the relevance of cellular senescence to the seCad production, A549 human lung epithelial cells were cultured in the presence of conditioned media (CM) of senescent or non-senescent mouse embryonic fibroblasts (MEF). The senescent cell-derived CM enhanced the seCad production in A549 cells (Figure 2E), supporting our notion that cellular senescence promotes the seCad production in a non-cell-autonomous manner presumably through SASP.

### seCad enhanced the invasive activity of B16-F10 cells

**Figure 3:**
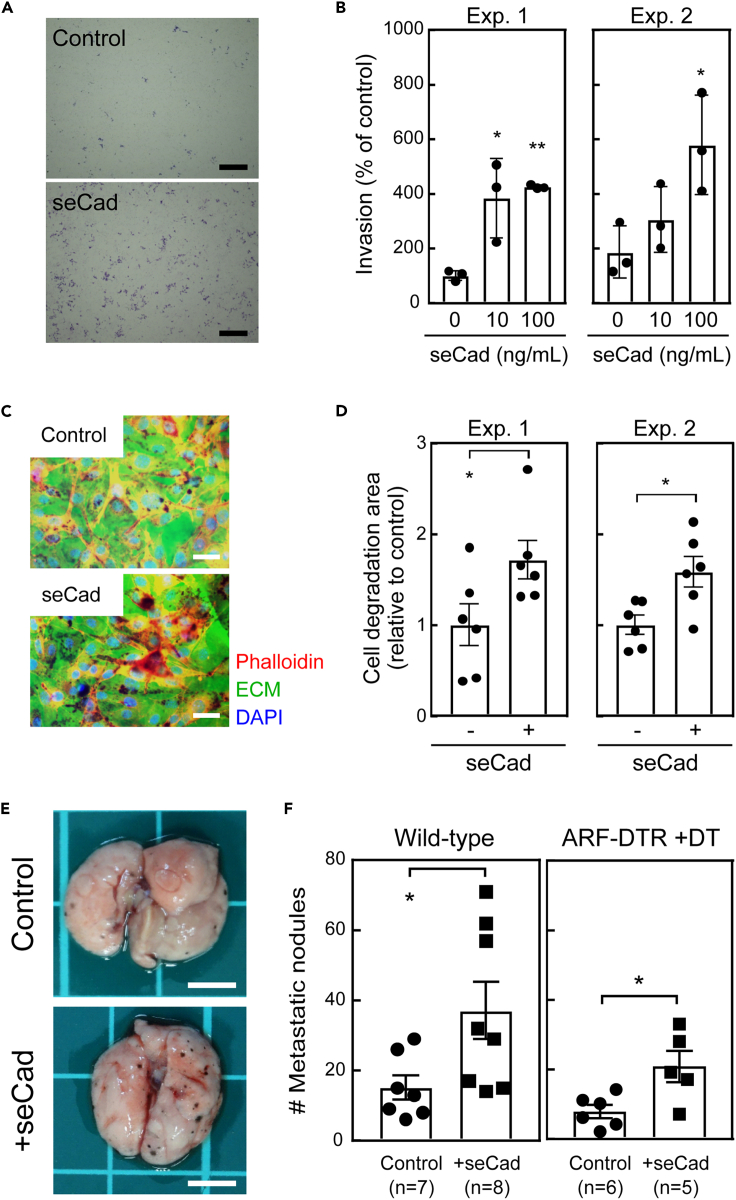
seCad enhanced the invasive features of B16-F10 melanoma cells (A) Representative images of the transwell invasion assay. B16-F10 cells were stimulated or unstimulated with recombinant seCad (100 ng/mL) for 48 hr. Scale bar, 500 μm. (B) The number of cells that migrated through the Matrigel-coated membrane were counted and relative changes to the average number detected in the control sample were plotted. The results of two independent experiments are shown. Data are presented as the mean value ±SD of triplicate samples. (C) Representative images of fluorescent dye-conjugated Gelatin zymography. Green, fluorescein-Gelatin; Red, TRITC-Phalloidin; Blue, DAPI. Scale bar, 50 μm. (D) The area of Gelatin degradation was measured. The degradation area relative to that of the control sample was calculated. Six randomly selected fields were analyzed in each sample. Values represent the mean ± SD. Results of two independent experiments are shown. (E) Representative images of lung tissues. B16-F10 cells were cultured in the presence or absence of seCad (100 ng/mL) for 24 hr and injected into the tail vein of wild-type mice. Lung metastasis was analyzed 2 weeks after the injection. Scale bar, 5 mm. (F) The number of tumor nodules in the left lung lobe was counted. B16-F10 cells treated or untreated with seCad were injected into wild-type (*left*) or ARF-DTR (*right*) mice. DT was administered to ARF-DTR mice prior to the B16-F10 injection. Data are presented as the mean value ±SEM in each sample. Data were analyzed by a one-way ANOVA and Tukey post-hoc analysis. Student's *t*-test was performed for the analysis of Gelatin zymography and the *in vivo* metastasis assay. ∗p < 0.05, ∗∗p < 0.01, and ∗∗∗p < 0.001. See also Figures S4 and S5.

Next, we investigated whether seCad, the production of which was dependent on p19^Arf^ expression in adult mice, could directly affect the invasive activity of B16-F10 cells. To this end, we first performed an *in vitro* invasion assay in the presence or absence of recombinant seCad, and found that the invasive activity of B16-F10 cells was enhanced in a seCad-dose-dependent manner (Figures 3A and 3B). Degradation of the proximal extracellular matrix (ECM) is a critical step for the invasion of tumor cells. To analyze whether seCad has an effect on the ECM-degrading activity of B16-F10 cells, we performed an *in situ* zymography analysis using a fluorescence dye-labeled ECM (Figure 3C). B16-F10 cells themselves exhibit a substantial ECM-degrading activity, which was further enhanced in the presence of seCad (Figure 3D). MMPs contribute to the ECM-degrading activity, and their activity and expression are associated with malignancy (Kessenbrock et al., 2010). In addition, seCad has been shown to induce the expression of several MMPs in human breast cancer cells (Nawrocki-Raby et al., 2003). Therefore, we investigated whether the expression of *Mmp* was affected by seCad in cultured B16-F10 cells. Among the *Mmp*s tested (*Mmp-2*, *7*, *9*, *10*, and *14*), the *Mmp-2* and *Mmp-14* mRNAs were reproducibly increased by seCad in B16-F10 cells (Figure S4A). Taken together, these results suggest that seCad promotes the invasive activity of B16-F10 cells through the enhancement of *Mmp* expression and ECM-degrading activity.

Next, we checked if the seCad-treated cells had a higher metastatic activity *in vivo*. B16-F10 cells were cultured in the presence or absence of seCad for 24 hr before intravenous injection. As shown in Figures 3E and 3F, we observed a significant increase in the number of metastatic nodules in mice transplanted with seCad-treated cells in wild-type animals. Similarly, seCad increased the B16-F10 metastasis in ARF-DTR mice treated with DT (Figure 3F, *right panel*), suggesting that seCad pretreatment was sufficient to enhance the metastasis of B16-F10 cells *in vivo*. Moreover, seCad did not affect the B16-F10 cell growth (Figure S5), and the increase in metastasis likely reflects their enhanced invasive activity. These results further support our hypothesis that seCad promotes the lung metastasis of B16-F10 cells by enhancing their invasive activity. Nonetheless, it is also possible that seCad modifies the host environments toward a setting that is more favorable for metastasis, in addition to acting directly on cancer cells.

### The PI3K pathway was involved in the seCad-mediated tumor cell invasion

**Figure 4:**
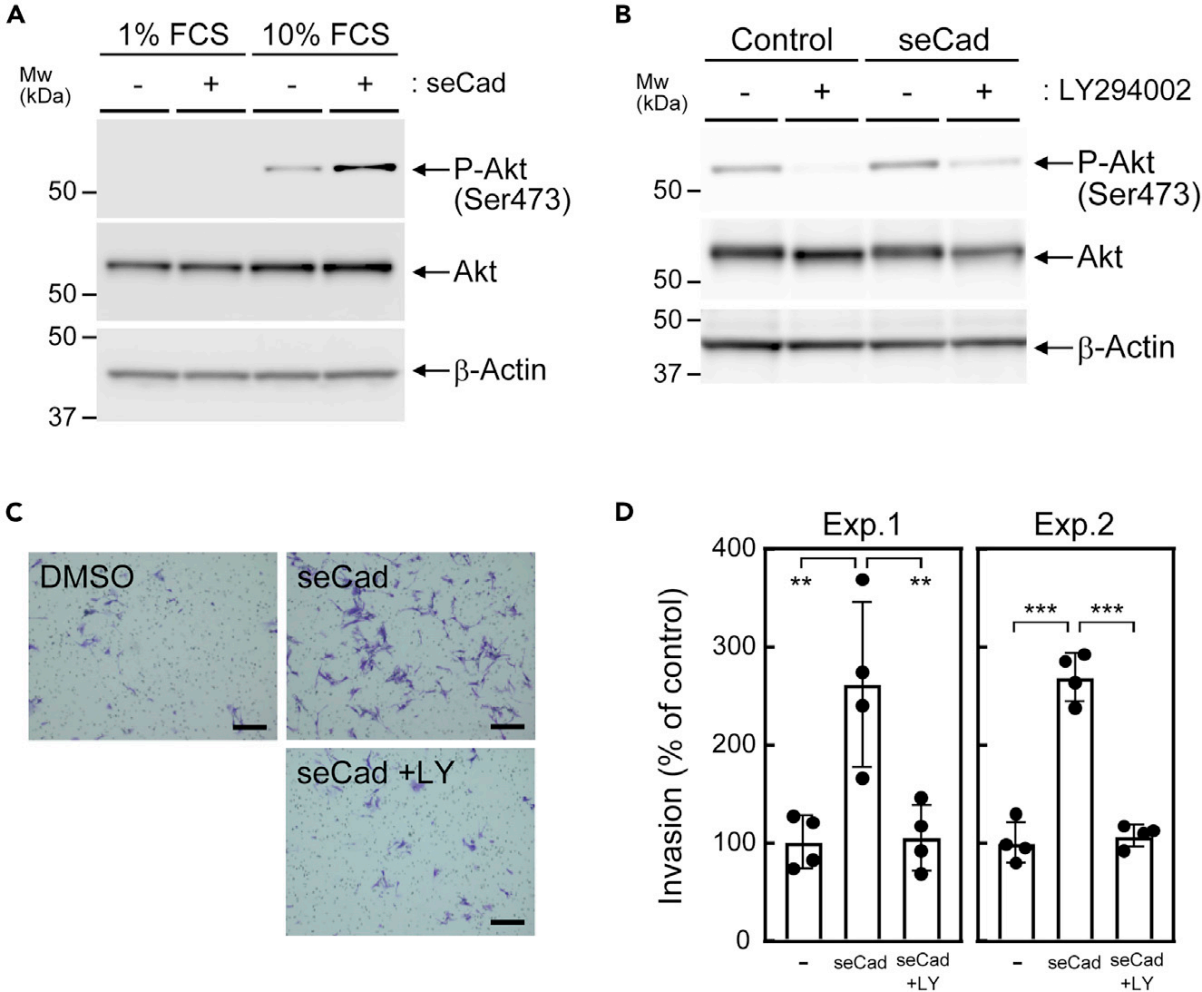
seCad enhanced serum-activated PI3K signaling (A) The expression of the indicated proteins was analyzed by immunoblotting. β-Actin was used as a loading control. (B) seCad induced Akt phosphorylation through PI3K. B16-F10 cells were cultured in the presence of LY294002 (LY, 20 μM) for 1 hr prior to the seCad stimulation. (C) Representative images of the transwell invasion assay. Cells were cultured in the presence or absence of seCad (100 ng/mL) together with LY for 48 hr. Scale bar, 200 μm. (D) The invasion activity of B16-F10 cells was inhibited by the PI3K inhibitor. Values represent the mean ± SD of quadruplicate samples. Results of two independent experiments are shown. Data were analyzed by one-way ANOVA and Tukey post-hoc analysis. ∗∗p < 0.01, and ∗∗∗p < 0.001. See also Figure S4.

In addition to the canonical E-cadherin signaling, seCad has been shown to activate growth-factor signaling by directly interacting and activating receptor tyrosine kinases (Brouxhon et al., 2014b; Inge et al., 2011). Hence, next we sought to determine if seCad could activate this signaling. Phosphorylation of Akt at Ser473, which is catalyzed predominantly by PI3K, was not detectable in B16-F10 cells cultured in a medium supplemented with 1% serum (Figure 4A), but was readily detectable when cells were cultured in the presence of 10% serum. The addition of seCad further enhanced the serum-induced Akt phosphorylation, although it had no noticeable effect in cells cultured under a low serum concentration, suggesting that seCad possesses activity to enhance the growth-factor-dependent PI3K signal, but does not activate the signal by itself. In contrast, Erk phosphorylation was detectable regardless of its serum concentration in B16-F10 cells, in which seCad did not cause a change (data not shown).

To confirm that seCad promotes Akt phosphorylation through PI3K, cells were treated with a specific inhibitor of PI3K (LY294002) before the seCad stimulation in the presence of 10% serum. The Akt phosphorylation was abolished by LY294002, and seCad hardly induced phosphorylation under the same condition (Figure 4B), corroborating that seCad acted through the PI3K to enhance the Akt phosphorylation. We next checked whether the PI3K is required to promote the invasive activity of B16-F10 cells. Inhibition of PI3K activity by LY294002 blunted the effect of seCad on the *in vitro* invasion assay (Figures 4C and 4D). Similarly, the induction of *Mmp-2* and *Mmp-14* expression was inhibited by LY294002 (Figure S4B). Thus, it is plausible that seCad directly acts on the B16-F10 cells to enhance their invasion activity and the PI3K signaling at least partly mediates this effect. Nonetheless, the Akt phosphorylation was barely detectable in the established B16-F10 tumor nodules in lung, and no change was observed by DT or seCad treatment (data not shown), suggesting that seCad signal plays a role in the early stage of metastasis.

### Inhibition of seCad suppressed the lung metastasis of B16-F10 cells

**Figure 5:**
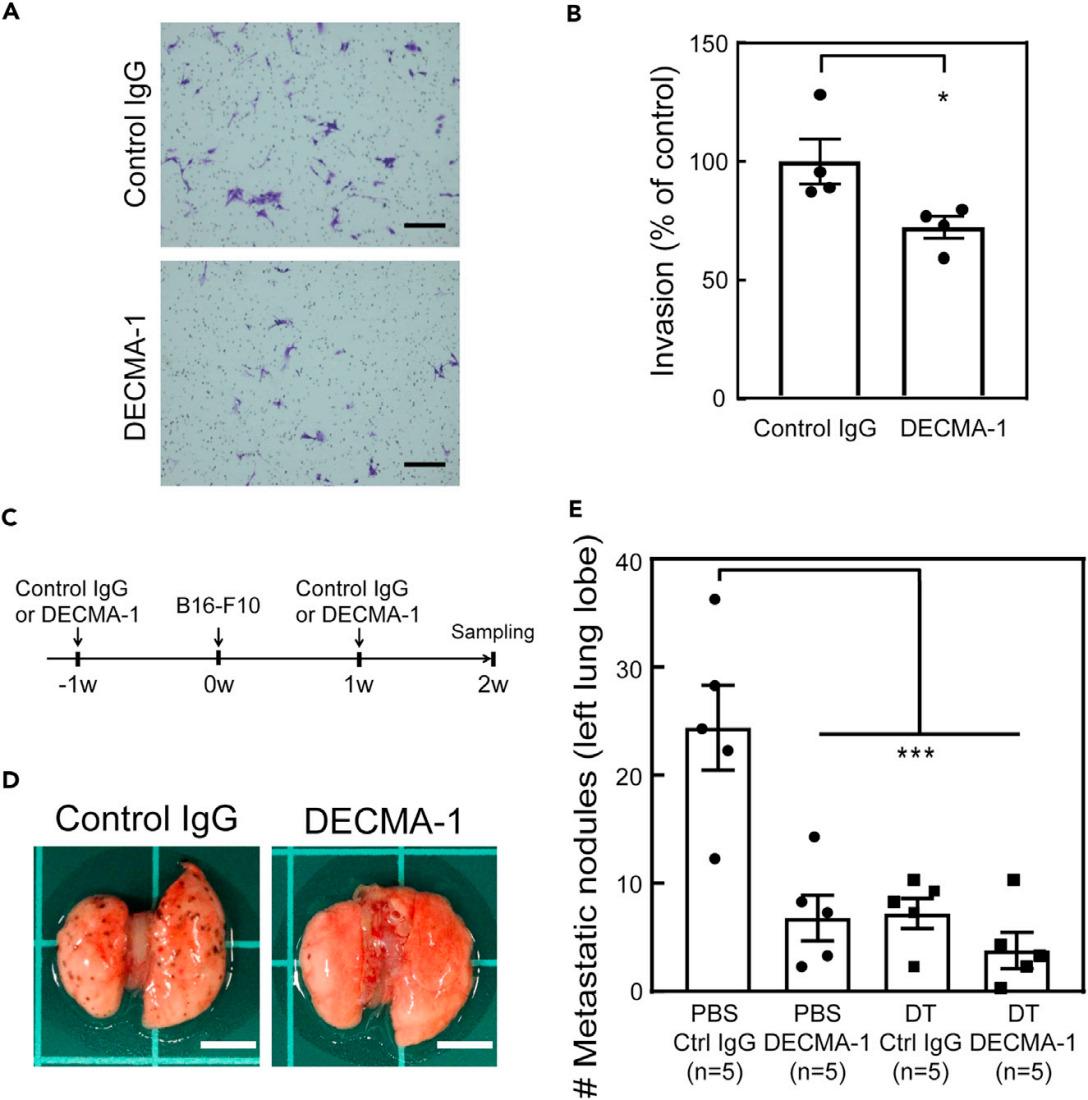
Inhibition of seCad suppressed the metastasis of B16-F10 cells to the lung (A) The transwell invasion assay was performed in the presence or absence of DECMA-1. Representative images are shown. Scale bar, 200 μm. (B) Inhibition of seCad suppressed the invasive activity of B16-F10 cells. Values represent the mean ± SD of quadruplicate samples. (C) Schematic chart of the experimental schedule. Twelve-month-old wild-type mice were intraperitoneally injected with control IgG or DECMA-1 prior to the intravenous injection of B16-F10 cells. Antibodies were re-injected 1 week after B16-F10 injection. (D) Representative images of lung tissues of wild type mice are shown. Scale bar, 5 mm. (E) The number of tumor nodules in ARF-DTR mice (left lobe) was counted. Data are presented as the mean values ±SEM. Data were analyzed by one-way ANOVA and Tukey post-hoc analysis. ∗p < 0.05 and ∗∗∗p < 0.001. See also Figure S6.

Next, we investigated if the inhibition of seCad had an effect on lung tumor metastasis. For this purpose, we used a neutralizing antibody against seCad, DECMA-1, which preferentially recognizes the soluble form of E-cadherin (Brouxhon et al., 2013, 2014a). Using DECMA-1, we first performed an *in vitro* invasion assay. We observed a slight but statistically significant reduction in the invasion activity in cells cultured in medium containing DECMA-1 (Figures 5A and 5B). This effect was not likely attributed to altered cell viability or growth, as DECMA-1 had no significant effect on these activities *in vitro* (Figures S6A–S6C).

To further validate the effect of seCad inhibition, an *in vivo* metastasis assay was performed. Mice were injected with control IgG or DECMA-1 before the intravenous injection of the B16-F10 cells (Figure 5C). As shown in Figures 5D, 5E, and S6D, there was a significant reduction in the number of metastatic nodules in lung tissues in both wild-type and ARF-DTR mice injected with DECMA-1 compared with those injected with control IgG. DECMA-1 had no additive effect on the B16-F10 metastasis in DT-treated ARF-DTR mice (Figure 5E), suggesting that senescent cells promote metastasis largely through seCad. Interestingly, the effect seemed to be much stronger than that obtained from *in vitro* experiments, which also suggested the possibility that seCad also acts on the tumor environments in addition to the direct effect on the B16-F10 cells. Inhibition of intact E-cadherin may also contribute to the reduced metastasis, because DECMA-1 could bind to the full-length E-cadherin protein as well.

### seCad induced genes associated with poor prognosis in human melanoma patients

**Figure 6:**
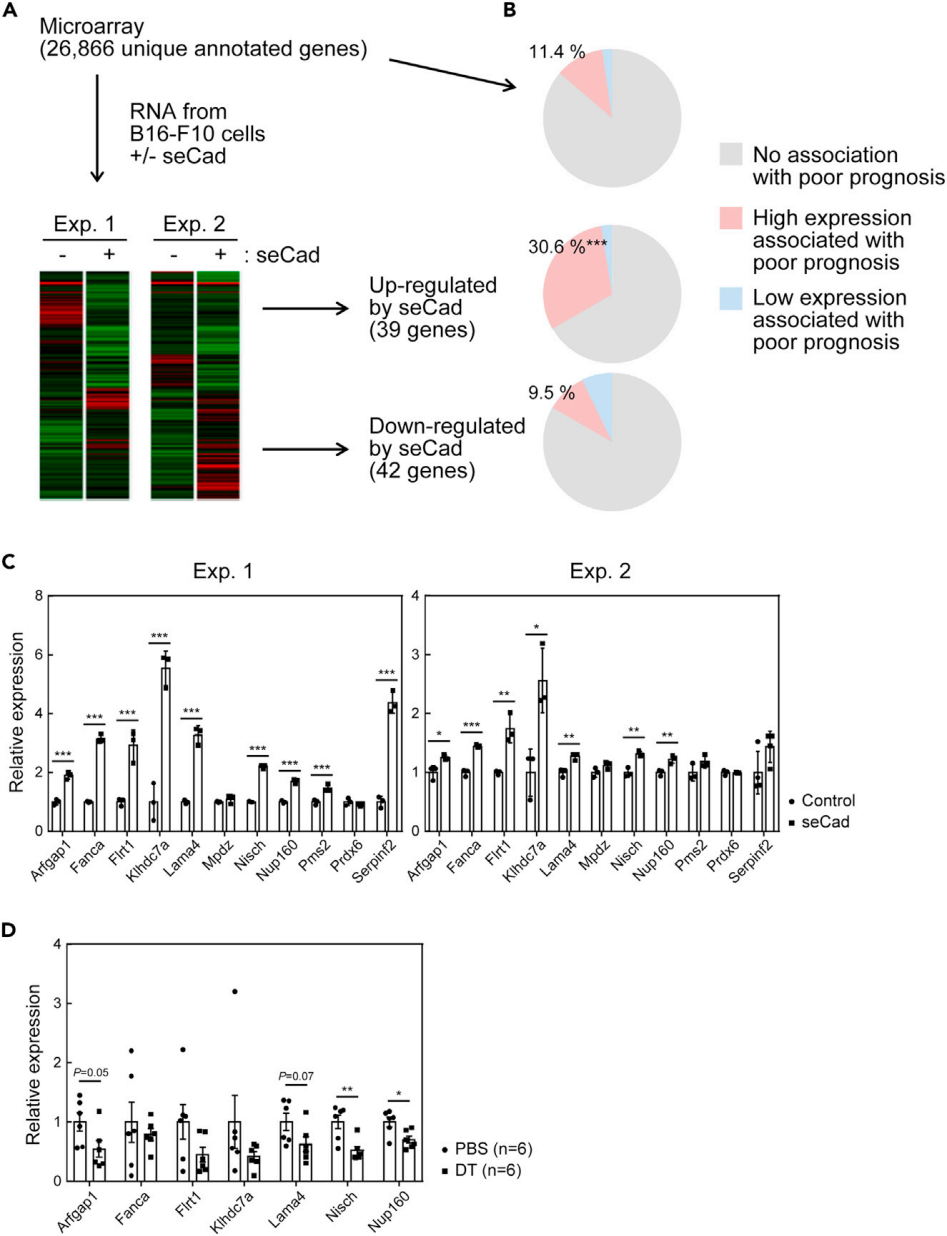
seCad induced genes associated with poor prognosis in melanoma patients (A) Schematic chart of the gene expression analysis. The RNA of B16-F10 cells treated or untreated with seCad was analyzed by a microarray containing 26,866 unique annotated genes. The heatmaps represent the results of two-independent experiments (genes with >2-fold change by seCad). A full list of the microarray data is available at the Gene Expression Omnibus database (accession number; GSE147154). (B) Total genes, genes upregulated or downregulated by seCad (>2-fold) were analyzed for their association with a poor prognosis in patients with melanoma using the PrognoScan database (http://dna00.bio.kyutech.ac.jp/PrognoScan/). The results are presented as pie charts. The number in each pie chart indicates the proportion of genes for which a high level of expression is associated with poor prognosis, which was analyzed by the chi-squared test. ∗∗∗p < 0.001. Full lists of genes are presented in the Tables S1 and S2. (C) Real-time PCR analysis of the genes for which an increased level of expression was associated with poor prognosis and that were induced by seCad. B16-F10 cells were cultured in the absence or presence of seCad (100 ng/mL) for 24 hr, and the expression of the indicated genes was analyzed. Values were normalized to the *Hprt* mRNA in each sample. The results of two independent experiments are shown. (D) in the B16-F10 lung nodules in PBS- or DT-treated ARF-DTR mice were collected and the expression of indicated mRNA was analyzed by real-time PCR. Student's *t*-test was performed for the comparison of each mRNA level in the control and seCad samples. Data represent the mean ± SD. ∗p < 0.05, ∗∗p < 0.01 and ∗∗∗p < 0.001. See also Tables S1 and S2.

To gain further insights into the roles of seCad in tumor pathology, we analyzed gene expression profiles. Total RNA isolated from B16-F10 cells cultured in the presence or absence of seCad was subjected to microarray analysis (Figure 6A). The results indicated that 39 and 42 genes were consistently up- or downregulated (>2-fold) by seCad, respectively, in B16-F10 cells in two independent experiments (Figure 6A and Table S1).

The microarray used in the analysis contained 26,866 uniquely annotated genes. We analyzed the association of human orthologs of those 26,866 genes with the prognosis of patients with melanoma using the PrognoScan database, and found that 11.4% of the genes showed an association between their high expression levels in cancer cells and poor prognosis (Figure 6B and Table S2). In contrast, an association between their low expression levels and poor prognosis was observed for only 2.25% of the genes. We performed a similar analysis on the genes that were upregulated or downregulated by seCad. Interestingly, we observed a substantial enrichment of the genes for which their high expression level was associated with poor prognosis among the upregulated genes (p < 0.001); however, no enrichment was detected for the downregulated genes. Expression of the upregulated genes for which their expression was associated with poor prognosis was independently validated by real-time PCR (Figure 6C). We further analyzed the expression of those genes in B16-F10 metastatic nodules in ARF-DTR mice. Among genes of which expression was upregulated by seCad in cultured B16-F10 cells in two independent experiments, expression of 4 genes including *Arfgap1*, *Lama4*, *Nisch* and *Nup160* was diminished in DT-treated ARF-DTR mice (Figure 6D). Taken together, these results imply that seCad induces a gene expression shift toward more-malignant melanoma.

### Increased serum seCad levels were associated with lung metastasis in patients with melanoma

**Figure 7:**
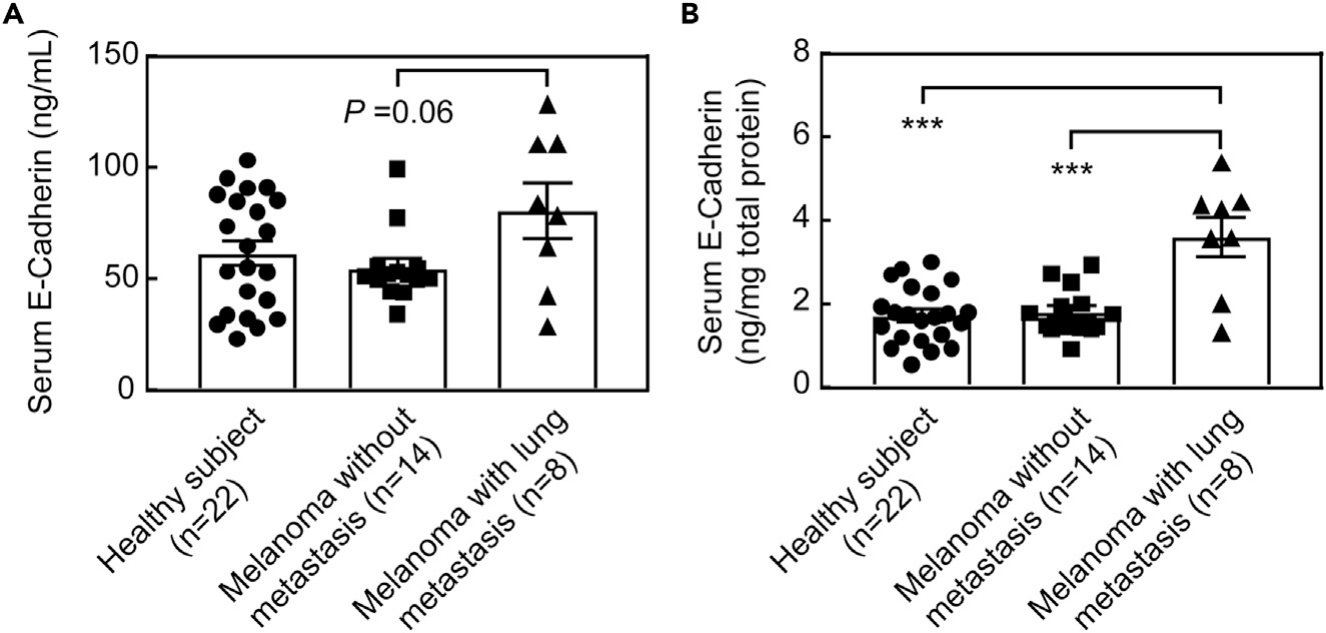
Serum seCad levels were increased in patients with melanoma with lung metastasis Detailed patient information is presented in Table S3. (A) Sera obtained from healthy subjects or patients with melanoma with no metastasis or lung metastasis were analyzed for seCad level. (B) Serum seCad levels were normalized to the protein amount in each sample. Data represent the mean values ±SEM in each group. Data were analyzed by one-way ANOVA and Tukey post-hoc analysis. ∗∗∗p < 0.001. See also Figure S7.

The results presented above suggest that seCad is involved in tumor metastasis in patients with melanoma. A previous study also reported an increased blood seCad level in patients with melanoma (Billion et al., 2006). Hence, we analyzed the blood seCad level in healthy subjects and patients with melanoma. The patients were categorized into two groups, i.e., subjects with no metastasis and subjects with lung metastasis (Table S3), and serum seCad levels were analyzed by ELISA. Although there was no significant difference in the serum seCad levels between healthy subjects and patients with melanoma without metastasis, a substantial increase in plasma seCad was observed in patients with melanoma with lung metastasis (Figure 7A). The difference was more evident when the seCad level was normalized to serum protein content (Figure 7B) or in the female samples (Figure S7). Collectively, these results suggest that serum seCad is associated with the metastasis of melanoma in human patients and is a potential diagnostic marker of malignant melanoma.

## Discussion

Aging is a major risk factor for cancer, and it also increases the incidence of melanoma and leads to poor prognosis in patients with melanoma (Tsai et al., 2010). Aging is associated with the accumulation of senescent cells. Although cellular senescence prevents malignant transformation, it can also promote cancer progression in a non-cell-autonomous manner through SASP (Ohtani, 2019). For instance, interleukin-6, which is a major component of SASP, exhibits potential to accelerate tumor cell proliferation (Di et al., 2014). However, the tumor-promoting effects of senescent cells are complicated and remain to be elucidated. We found that the elimination of p19^Arf^-expressing senescent cells from lung tissues resulted in a significant suppression of melanoma metastasis in mice. Our results are consistent with a previous study that was performed using p16-3MR mice (Demaria et al., 2017). We further investigated the underlying mechanism and found that seCad can at least partly mediate the senescence-enhanced metastasis of melanoma to the lung. The production of seCad was dependent on the p19^Arf^-expressing senescent cells in adult mice. Multiple proteinases can mediate the ectodomain shedding of E-cadherin, and Adam10 is one of the most important enzymes for this process (Maretzky et al., 2005). Thus, Adam10 is likely responsible for the senescence-dependent ectodomain shedding of E-cadherin, as its expression was significantly decreased in the DT-treated ARF-DTR lung tissues, although other enzymes, such as Mmp, may also contribute to this process (Hashimoto et al., 2016). Although the p19^Arf^-expressing senescent cells are predominantly detected in the lung and ablated by DT in adult ARF-DTR mice (Hashimoto et al., 2016; Mikawa et al., 2018, 2020), the possibility that the systemic senescent cells or senescence in other tissue may also contribute to the seCad production. Tissue-specific senescent cell ablation system needs to be established to address this question.

Although SASP was initially considered to elicit the clearance of senescent cells, it has become apparent that SASP can also promote tumor progression and metastasis. Senescent cells create an immunosuppressive environment, thereby promoting tumor growth in a hepatocellular carcinoma model (Eggert et al., 2016); moreover, Loo et al. also demonstrated that senescent stellate cells suppressed the anti-tumor response through SASP (Loo et al., 2017). Furthermore, senescent cells drive angiogenesis by secreting the vascular endothelial growth factor, which promotes metastasis (Coppe et al., 2006; Oubaha et al., 2016). Thus, while the data presented above imply that seCad acts directly on the cancer cells to enhance their invasive activity, it is quite reasonable to assume that seCad and other factors that are regulated by cellular senescence also affect the cancer environment and the properties of the cancer cells. So far, we did not observe any detectable change in the dynamics of immune cells including B cells, T cells, and macrophages in lung after the senescent cell elimination in ARF-DTR mice (data not shown). More comprehensive analysis will be needed to clarify the effects of seCad and senescent cells on immune cell function and tumor environment.

Our antibody array also revealed that the macrophage inflammatory protein-1 gamma (Mip-1γ) was inversely associated with lung senescence. However, Mip-1γ promotes cancer invasion rather than suppression (Kitamura et al., 2007), which further complicates the SASP-mediated cancer regulation. Nevertheless, in our model, seCad likely plays a key role in the senescence-dependent metastasis because its antibody-mediated inhibition significantly reduced the lung metastasis of B16-F10 cells.

Although seCad directly enhanced the invasive activity of B16-F10 cells, questions still remain regarding its underlying molecular mechanism. It is unlikely seCad transduces the signal through the homotypic binding as E-cadherin protein is not detected in B16-F10 cells (data not shown). seCad has been shown to activate EGFR and IGFR (Brouxhon et al., 2014b; Inge et al., 2011). We used a receptor tyrosine kinase array to analyze the effect of seCad on growth factor signaling, but did not observe any changes in receptor phosphorylation on the array (data not shown). Nevertheless, our data indicate that seCad enhances the growth factor-dependent PI3K activation. Although the manner in which seCad modulates growth factor signaling remains to be elucidated, the PI3K activity seems essential for *Mmp* induction and invasion *in vitro*. In contrast, the relevance of PI3K in *in vivo* tumor metastasis is also unknown. We have not been able to detect Akt phosphorylation in the metastatic nodules formed by B16-F10 in the lung, suggesting that the enhanced PI3K activity is only required during the early stage of metastasis.

Our data also pointed out the possibility that seCad plays a role in melanoma metastasis in humans. In this regard, Nissen and colleagues reported is the presence of a correlation between serum seCad and S100 tumor marker levels in patients with melanoma (Billion et al., 2006). Although we did not observe a difference between healthy subjects and patients with melanoma without metastasis, significantly higher serum seCad levels were detected in the patients with melanoma with distant metastasis, suggesting that seCad is a potential biomarker for prognosis prediction. Although whether the increased seCad level is a cause or a consequence of cancer progression remains to be clarified, our gene expression profiling analysis strongly suggests that seCad can cause a shift in gene expression to a pattern that is associated with poor prognosis in humans. However, because the overlap between seCad-treated B16-F10 cells and malignant human melanoma was limited, other senescence-dependent factor(s) may also contribute to the malignancy. Moreover, serum seCad has been shown to be increased in many types of cancer (De Wever et al., 2007). Therefore, it is feasible that seCad has an effect on other types of cancer similar to that observed in melanoma.

Metastasis is the main cause of death among patients with melanoma. Our data, together with the data from other groups, indicated that the elimination of senescent cells is effective in preventing metastasis. In humans, senolysis can be carried out by senolytic drugs that specifically target senescent cells (Kirkland and Tchkonia, 2017). However, a recent report that used a mouse model to target senescent cells revealed that the elimination of cells with a high level of p16^Ink4a^ had adverse effects, including reduced health span and the induction of fibrosis in multiple tissues because of the incapability of replacement of Cd31-positive endothelial cells (Grosse et al., 2020). Thus, the usage of a senolytic approach might not be necessarily ideal for therapy; in contrast, such adverse effects have not been observed for dasatinib/quercetin (Grosse et al., 2020; Justice et al., 2019). To circumvent the adverse effects of senolysis, the neutralization or inhibition of seCad by specific antibodies or small compounds may be a useful approach for the inhibition of the metastasis of melanoma.

### Limitations of the study

We focused on lung senescent cells in ARF-DTR mice. However, the involvement of systemic senescent cells in seCad production and melanoma metastasis has not been excluded in this study as described in the discussion. Tissue-specific ablation of senescent cells needs to be established to address this issue.

Although seCad levels correlated with lung metastasis in patients with melanoma, cellular senescence in those patients could not be analyzed due to the unavailability of lung samples.

## STAR★Methods

### Key resources table

**Table:** 

REAGENT or RESOURCE	SOURCE	IDENTIFIER
**Antibodies**		

Rabbit polyclonal anti-E-Cadherin	GeneTex	Cat#GTX100443; RRID: AB_10729586
Rabbit monoclonal anti-Akt (pan)	Cell Signaling Technology	Cat#4691; RRID: AB_915783
Rabbit monoclonal anti-phospho-Akt (ser473)	Cell Signaling Technology	Cat#4060; RRID: AB_2315049
Rabbit monoclonal anti-β-actin (HRP conjugate)	Cell Signaling Technology	Cat#12620; RRID: AB_2797972
Rabbit polyclonal anti-p16INK4a	This paper	N/A
Rat monoclonal anti-p19ARF	Santa Cruz Biotechnology	Cat#sc-32748; RRID: AB_628071
Alexa Fluor 647-conjugated anti-rat IgG	Jackson ImmunoResearch	Cat#712-606-150; RRID: AB_2340695
Alexa Fluor 488-conjugated anti-rabbit IgG	Jackson ImmunoResearch	Cat#711-545-152; RRID: AB_2313584
CD324 monoclonal antibody (clone DECMA-1)	Thermo Fisher Scientific	Cat#16-3249-85; RRID: AB_10735370
Rat IgG1κ Isotype control (clone eBRG1)	Thermo Fisher Scientific	Cat#16-4301-81; RRID: AB_470153

**Biological samples**		

Human sera	ProteoGenex	Table S3

**Chemicals, peptides, and recombinant proteins**		

Diphtheria toxin	Sigma-Aldrich	Cat#D0564
VivoGlo™ Luciferin, In Vivo Grade	Promega	Cat#P1043
Recombinant Mouse E-Cadherin Protein	R&D systems	Cat#8875-EC-050
LY294002	Chemscene	Cat#CS-0150; CAS: 154447-36-6

**Critical commercial assays**		

Mouse Cytokine Antibody Array	Abcam	Cat#Ab193660
Mouse E-cadherin ELISA Kit	Biosensis	Cat#BEK-2185-1P
QCM™ Gelatin Invadopodia Assay	Millipore	Cat#ECM670
Cellular Senescence Detection Kit - SPiDER-βGal	Dojindo	Cat#SG03
Human E-Cadherin Quantikine ELISA Kit	R&D systems	Cat#DCADE0
NucleoSpin RNA	Macherey-Nagel	Cat# 740955.10
PrimeScript RT Reagent Kit with gDNA Eraser	Takara Bio	Cat# RR047A

**Deposited data**		

Microarray data	This paper	GEO: GSE147154

**Experimental models: Cell lines**		

Mouse: B16-F10	ATCC	Cat#CRL-6475; RRID: CVCL_0159
Human: A549	ATCC	Cat#CCL_185; RRID: CVCL_0023

**Experimental models: Organisms/strains**		

Mouse: ARF-DTR	Hashimoto et al. (2016)	N/A
Mouse: C57BL/6J	The Jackson Laboratory	RRID: IMSR_JAX:000664

**Software and algorithms**		

GraphPad Prism7	GraphPad	RRID: SCR_002798
PrognoScan	PrognoScan	http://www.prognoscan.org/

### Resource availability

#### Lead contact

Further information and requests for resources and reagents should be directed to and will be fulfilled by the lead contact, Masataka Sugimoto (msugimot@ncgg.go.jp).

#### Materials availability

All unique reagents in this study are available from the lead contact with a completed Material Transfer Agreement.

### Experimental model and subject details

All animal experiments were approved by the National Center for Geriatrics and Gerontology Animal Ethics Committee. Two- to 6-month-old ARF-DTR mice (Hashimoto et al., 2016) and their wild-type littermates in the C57BL/6J (RRID: IMSR_JAX:000664) background were maintained under specific pathogen-free conditions with a 12 h dark/light cycle, constant temperature, and *ad liberum* access to food (CE-2, Japan CLEA) and water. Female mice were used in all experiments except for Figure 3F, in which sex was randomized. No sex difference was observed in the effect of senescent cell elimination on the lung metastasis of melanoma cells. To eliminate p19^Arf^-expressing cells, diphtheria toxin (D0564, 50 μg/kg of body weight; Sigma-Aldrich) was intraperitoneally injected twice with a 2-week interval. The BALF was prepared with 1 mL of PBS per mouse, and cells and debris were removed by mild centrifugation.

For lung metastasis assay, B16-F10 cells (500,000 cells in 0.2 mL of PBS) were injected into the mouse tail vein (Fidler, 1973). Two weeks later, mice were euthanized and the left lung lobe (Figures 1 and 3) or both left and right lung lobes (Figure 5) was dissected under a stereomicroscope to count the number of metastatic nodules. To neutralize seCad *in vivo*, DECMA-1 (1 mg/kg of body weight; 16-3249-85, ThermoFisher Scientific) or control IgG (16-4301-81, ThermoFisher Scientific) was intraperitoneally injected twice with a 2-week interval into the wild-type mice.

For the measurement of subcutaneous tumor size and lymph node metastasis, 400,000 B16-F10 cells were subcutaneously injected. Tumor size and inguinal lymph node metastasis were analyzed 2 weeks after the tumor injection.

### Method details

#### *In vivo* imaging

ARF-DTR mice were anesthetized using 2% isoflurane (099-06571, Fuji Film) and 150 mg/kg body weight of luciferin (P1043, Promega) was intraperitoneally injected (Kawaguchi et al., 2021). Luciferase activity was monitored using the IVIS imaging system (Lumina II, Perkin Elmer).

#### Antibody array and immunoblotting

BALF samples were prepared from three mice in each group, pooled, and subjected to a Mouse Cytokine Antibody Array (ab193660, Abcam) according to the manufacturer’s instructions. Pooled BALF containing 100 μg of protein was used for each membrane. Signals were detected on an Amersham Imager 680 instrument (Cytiva) and analyzed by Image J.

For immunoblotting, lysates were prepared using RIPA buffer (10 mM Na-phosphate, pH 7.2, 150 mM NaCl, 2 mM EDTA, 0.1% SDS, 1% Na-deoxycholate, and 1% NP-40). Proteins were separated by SDS−PAGE, transferred to a PVDF membrane, and were detected using antibodies to E-cadherin (GTX100443, GeneTex), β-Actin (#12620, Cell Signaling Technology), phospho-Akt (ser473) (#4060, Cell Signaling Technology), and Akt (#4691, Cell Signaling Technology).

#### B16-F10 invasion assay and zymography

B16-F10 cells were maintained in Dulbecco’s Modified Eagle’s Medium (DMEM) supplemented with 10% fetal bovine serum (FBS) and penicillin/streptomycin. For proliferation assay, 200,000 cells were seeded in a cell culture dish (diameter, 6 cm) in the absence or presence of recombinant mouse E-cadherin (8875-EC-050, R&D systems). The number of cells was counted every 24 h.

For invasion assay, 100,000 cells suspended in a serum-free medium with or without recombinant E-cadherin were placed in the upper chamber of Matrigel (356234, Corning)-coated transwell cell culture inserts with a size of 8.0 μm (353097, Corning). The inserts were placed in a multi-well cell culture dish containing a complete medium with 10% serum. After 48 h of cell culture, cells in the upper compartment of transwell inserts were removed, and the cells that had migrated to the lower compartment were stained using Diff-Quik (16920, Sysmex).

*In vitro* zymography was performed using a QCM™ Gelatin Invadopodia Assay Kit (CM670, Millipore), according to the manufacturer’s instructions. B16-F10 cells were cultured on a Fluorescein-Gelatin-coated chamber slide for 48 h. Samples were stained with TRITC-Phalloidin and DAPI. Fluorescence images were acquired with a fluorescence microscope (Keyence) and analyzed using Image J. Cell area (TRITC-positive) to degradation area (Fluorescein-negative) ratios were calculated.

#### Enzyme-linked immunosorbent assay (ELISA)

Mouse BALF and plasma E-cadherin levels were analyzed using a Mouse E-cadherin ELISA Kit (BEK-2185-1P, Biosensis). Human sera (healthy subjects and melanoma patients, information including age, gender and pathology are listed in Table S3) were purchased from ProteoGenex, and E-cadherin levels were analyzed using a Quantkine® ELISA Human E-Cadherin. Data were normalized to the protein content in each sample.

#### SeCad measurement in A549 cells

Embryonic fibroblasts from C57BL/6J mice (MEFs) were maintained in Dulbecco’s Modified Eagle’s Medium (DMEM) supplemented with 10% fetal bovine serum (FBS) supplemented with non-essential amino acids and gentamycin, and cultured on a 3T3 protocol. Conditioned media obtained from non-senescent and senescent MEFs were diluted with equal volume of fresh medium and stored at −80°C until use.

A549 human lung epithelial cells (RRID: CVCL_0023) were seeded in 6-well plates. When cells reached to confluent, media were replaced by conditioned media of MEFs and cultured for 48 h. Culture supernatants were cleared by centrifugation and the E-cadherin levels were determined by ELISA for human E-cadherin.

#### Immunohistochemistry and SA β-gal staining

Bacterially produced full-length mouse p16^Ink4a^ was used to immunize rabbits, and antibodies were affinity purified. Frozen lung sections (8 μm in thickness) were immunostained using p19^Arf^ (1:100 dilution, sc-32748, Santa Cruz Biotechnology) and p16^Ink4a^ antibodies. Sections were visualized using Alexa Fluor 647-conjugated anti-rat IgG (1:1000 dilution, 712-606-150, Jackson ImmunoResearch) and Alexa Fluor 488-conjugated anti-rabbit IgG (1:1000 dilution, 711-545-152, Jackson ImmunoResearch). Sections were counterstained with DAPI. SA β-gal staining was performed using a Cellular Senescence Detection Kit—SPiDER βGal (SG03, Dojindo).

#### Real-time PCR

Total RNA was reverse transcribed using the PrimeScript RT Reagent Kit with gDNA Eraser (RR047A, Takara BIO Inc.). Real-time PCR was carried out on a CFX Connect Real Time System (BIO RAD) using the following primers. *Adam10*, 5′−GTGCCAAACGAGCAGTCTCA−3′ (sense) and 5′−ATTCGTAGGTTGAACTGTCTTCC−3′ (antisense); *Arfgap1*, 5′−AACACAGTGCCACCTCAGAA−3′ (sense) and 5′−ACCCTCCTTTGCTGCAGAT−3′ (antisense); *Fanca*, 5′−AATGACAGACCCGACCCAAT−3′ (sense) and 5′−GGAAGGAAGGACACTGAGCT−3′ (antisense); *Flrt1*, 5′−TGGCTGGGATTATTGGTGGT−3′ (sense) and 5′−CCCCTGTTGTAGACCCTCTC−3′ (antisense); *Gapdh*, 5′−AATGGTGAAGGTCGGTGTG−3′ (sense) and 5′−GAAGATGGTGATGGGCTTCC−3′ (antisense); *Hprt*, 5′−GGGGGCTATAAGTTCTTTGC−3′ (sense) and 5′−TCCAACACTTCGAGAGGTCC−3′ (antisense); *Klhdc7a*, 5′−ACCACATCTCACCCAGGTTT−3′ (sense) and 5′−ACAGGCAAGGTCAAGACGG−3′ (antisense); *Lama4*, 5′−CTCCTGCCTGATGTAGAGGG−3′ (sense) and 5′−CTCATGTTGTGGGCTTGCTC−3′ (antisense); *Mmp-2*, 5′−GGACAAGTGGTCCGCGTAAA−3′ (sense) and 5′−CCGACCGTTGAACAGGAAGG−3′ (antisense); *Mmp-14*, 5′−CAGTATGGCTACCTACCTCCAG−3′ (sense) and 5′−GCCTTGCCTGTCACTTGTAAA−3′ (antisense); *Mpdz*, 5′−TTGTCTTGCTTTCACTGGGC−3′ (sense) and 5′−CCTCGGTCTAGCGTAATGGT−3′ (antisense); *Nisch*, 5′−GGCCCTGTGACTGCTATCAT−3′ (sense) and 5′−ATCCACAACTCGCAGTCCAT−3′ (antisense); *Nup160*, 5′−AGTTGTTGTCTCATGGAGTTCC−3′ (sense) and 5′−TCAGAGACCAAATCCACAGCT−3′ (antisense); *Pms2*, 5′−TTCATTTCACAGTGCACGCA−3′ (sense) and 5′−CAAATGGGTACTGATGCCGG−3′ (antisense); *Prdx6*, 5′−CGCCAGAGTTTGCCAAGAG−3′ (sense) and 5′−GCAACTTTTCCGTGGGTGTT−3′ (antisense); *Serpinf2*, 5′−GCTGCCTAAACTCCATCTGC−3′ (sense) and 5′−ACACCACCAGATTCTGCTCA−3′ (antisense).

#### Microarray

Total RNA was extracted from B16-F10 cells cultured in the presence or absence of 100 ng/mL of seCad for 24 h using NucleoSpin RNA (Macherey-Nagel), according to the manufacturer’s instructions. A cyanine3-labeled RNA probe was prepared using the Low Input Quick Amp Labeling Kit (Agilent Technologies) and hybridized to the SurePrint G3 Mouse GE v2 8×60K Microarray (Agilent Technologies). Images were obtained using the SureScan Microarray Scanner (Agilent Technologies) and analyzed with the Agilent Feature Extraction (Agilent Technologies). Full data are available from the Gene Expression Omnibus database of the National Center for Biotechnology Information (accession number, GSE147154).

### Quantification and statistical analysis

A one-way ANOVA was performed for the comparison of more than two sets of data. When the statistical model was proven to be significant, differences between combinations of the two groups were analyzed using a Tukey−Kramer test. A two-tailed unpaired Student’s *t*-test was used for the comparison of two sets of experimental data. Data were analyzed by GrapgPad Prism 7.0 (GraphPad Software Inc.). Significance was represented by asterisks as follows: ∗p < 0.05, ∗∗p < 0.01, and ∗∗∗p < 0.001. No statistical method was used to select the sample size.

## Data Availability

This study did not generate code. The microarray data have been deposited at the National Center for Biotechnology Information (Gene Expression Omnibus database; accession number, GSE147154) and are publicly available as of the date of publication. Any additional information required to reanalyze the data reported in this paper is available from the lead contact upon request.

## Supplemental information

Document S1. Figures S1–S7

Table S1. List of genes that were up- or downregulated (>2-fold) by seCad, related to Figure 6

Table S2. List of genes associated with poor prognosis in patients with melanoma, related to Figure 6

Table S3. Patient information, related to Figure 7
