# Supplementary material for: Cellular senescence promotes cancer metastasis by enhancing soluble E-cadherin production
Source: iScience. 2021 Aug 24;24(9):103022. doi: 10.1016/j.isci.2021.103022 (PMC8426284; doi:10.1016/j.isci.2021.103022)
Supplement: Document S1. Figures S1–S7 [file mmc1.pdf]

iScience, Volume *24*

Supplemental information

Cellular senescence promotes cancer metastasis by enhancing soluble E-cadherin production

Koichiro Kawaguchi, Kaori Komoda, Ryuta Mikawa, Azusa Asai, and Masataka Sugimoto

Figure S1, relate to Figure 1

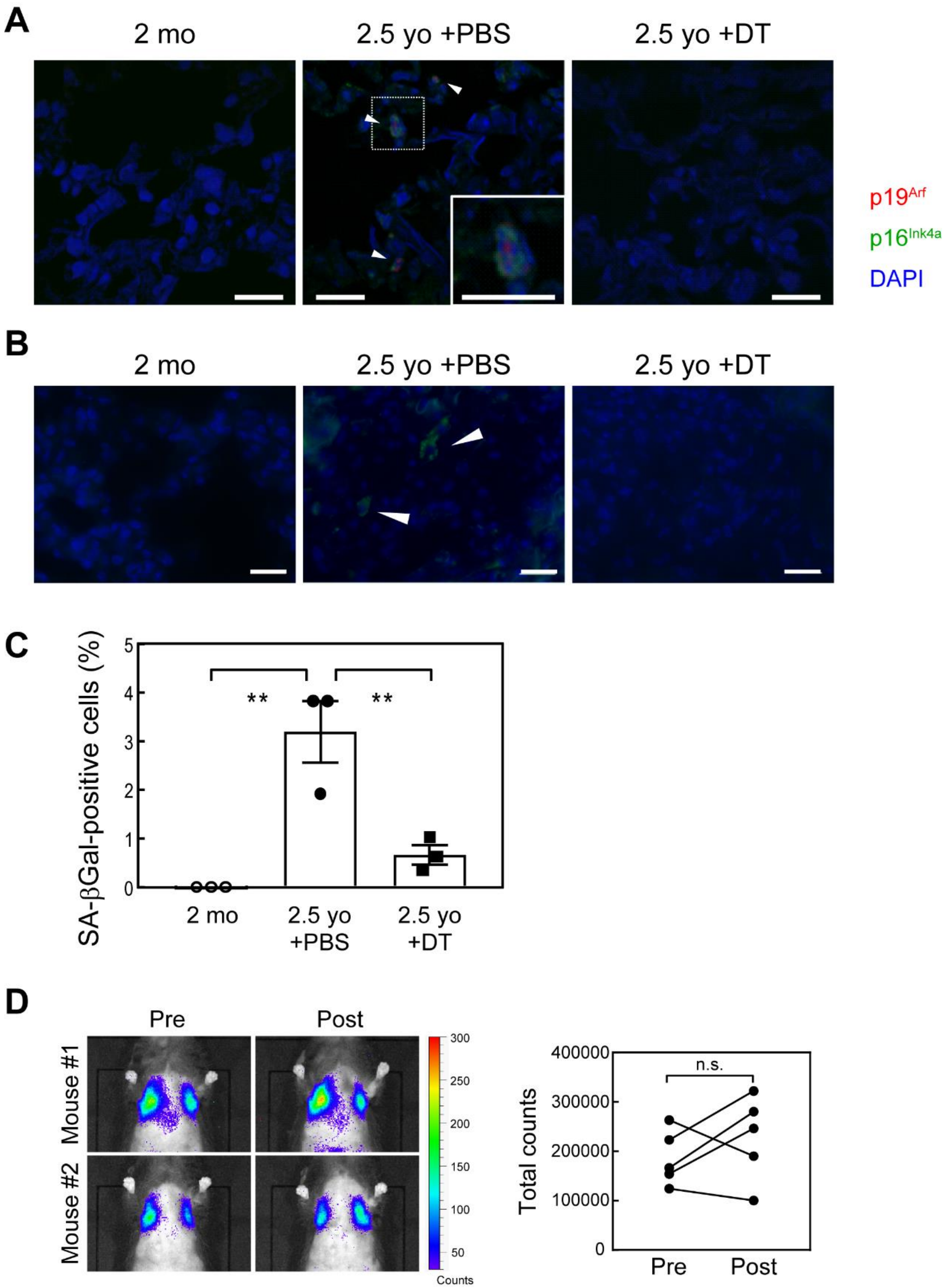

Figure S1. p19^{Arf}-expressing cells exhibited senescent cell features. (**A**) Representative images of p19^{Arf} and p16^{Ink4a} immunostaining in ARF-DTR lung. Frozen lung sections were prepared from ARF-DTR mice of the indicated age. The arrowheads indicate p19^{Arf}/p16^{Ink4a} double-positive cells. (**B**) Senescence-associated β-galactosidase in an ARF-DTR mouse lung. Frozen lung sections were stained for SA β-gal. Representative images are shown. The arrowheads indicate SA β-gal-positive cells. (**C**) The number of β-galactosidase-positive cells was counted. At least four randomly selected fields were used for quantification in each sample. Data are presented as the mean values ± SEM in each sample. Data were analyzed by one-way ANOVA and Tukey post-hoc analysis. **$P < 0.01$. Scale bar, 20 µm. (**D**) *Left*, Representative images of *in vivo* luciferase imaging were presented (*left*). Images were obtained prior to and 14 days post B16F10 injection. *Right*, Luciferase activity in chest region was measured. Changes in the luciferase activity in each mouse were plotted. Data were analyzed by paired *t*-test. n.s.: not significant.

Figure S2, relate to Figure 2

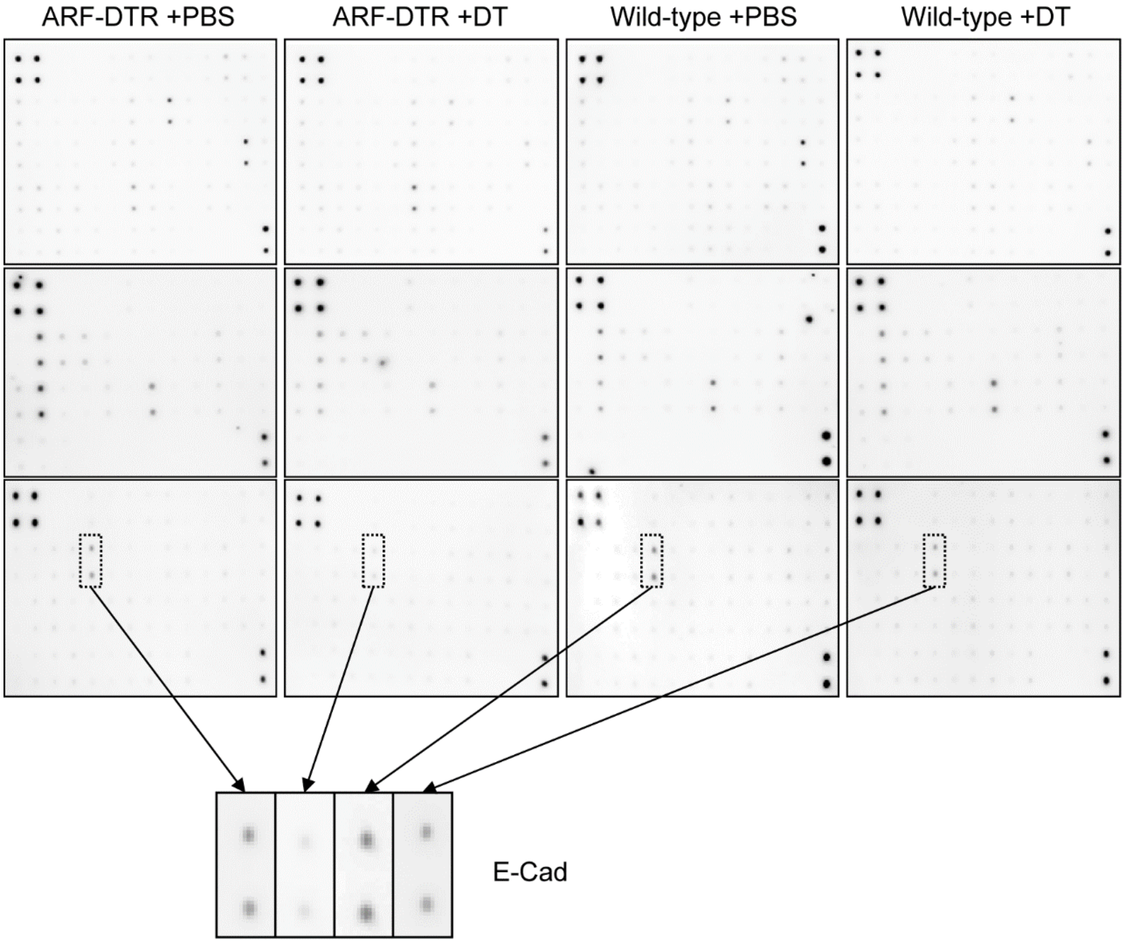

Figure S2. Screening of senescence-dependent humoral factors in BALF. The BALF of ARF-DTR or wild-type mice treated with PBS or DT was subjected to an antibody array analysis. BALF samples were prepared from four mice in each group and pooled before analysis. The area surrounded by a dotted line in each sample represents E-cadherin.

Figure S3, relate to Figure 2

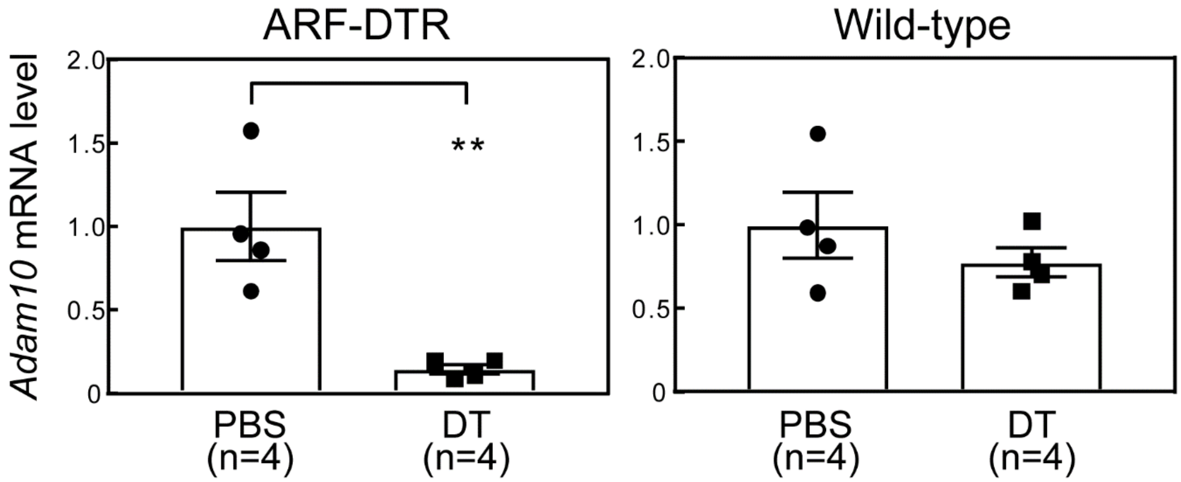

Figure S3. Possible involvement of Adam10 in senescence-dependent seCad production. Real-time PCR analysis of the *Adam10* mRNA in ARF-DTR and wild-type lung tissues of ARF-DTR or wild-type mice treated or untreated with DT. Values were normalized to *Gapdh* in each sample. Data are presented as the mean values ± SEM in each group. Data were analyzed by Student's *t*-test. $^{**}P < 0.01$.

Figure S4, relate to Figures 3 and 4

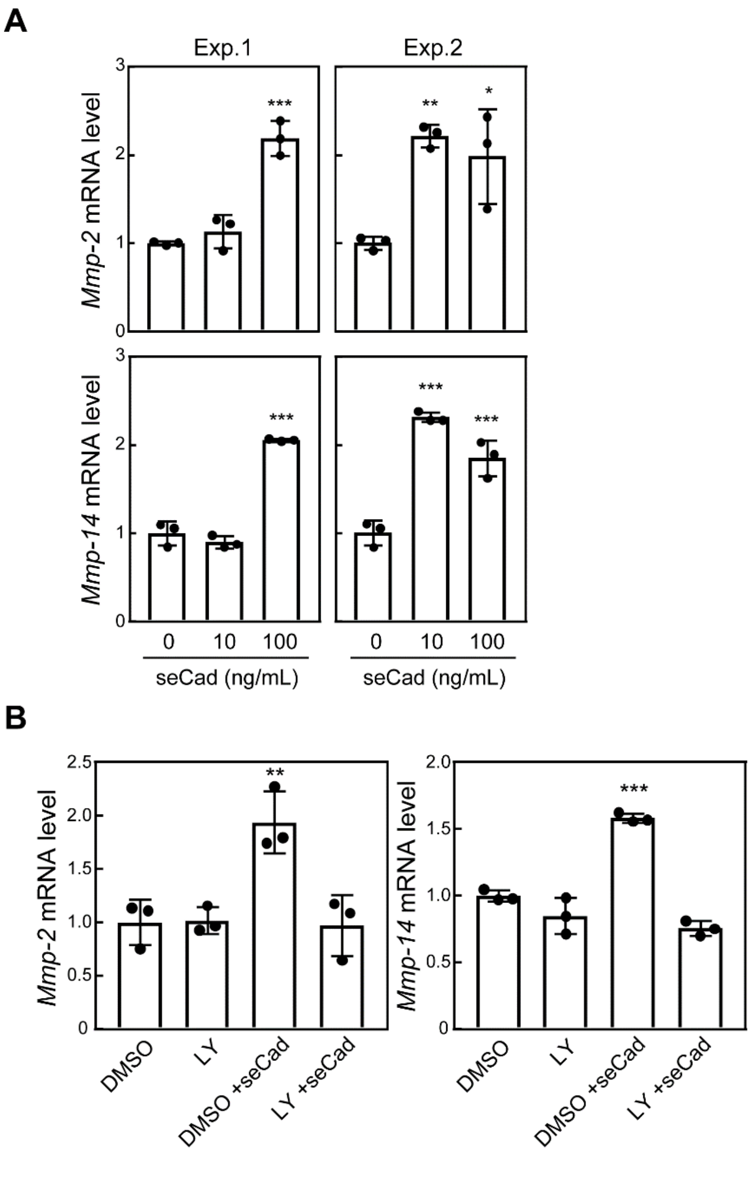

Figure S4. seCad induced *Mmp* expression in melanoma cells through PI3K. (**A**) B16-F10 cells were cultured in the presence of seCad (0, 10, and 100 ng/mL) for 24 h, and the expression of *Mmp-2* and *-14* was analyzed by real-time PCR. The results of two independent

experiments are shown. (**B**) Cells were cultured in the presence of seCad together with a PI3K inhibitor. The PI3K inhibitor was added 1 h prior to seCad stimulation (100 ng/mL). mRNA was extracted 6 h after seCad addition, and the expression of the indicated genes was analyzed by real-time PCR. Values were normalized to the *Hprt* mRNA in each sample. Dara are presented as the mean values ± SD. Data were analyzed by one-way ANOVA and Tukey post-hoc analysis. *$P < 0.05$, **$P < 0.01$, and ***$P < 0.001$.

Figure S5, relate to Figure 3

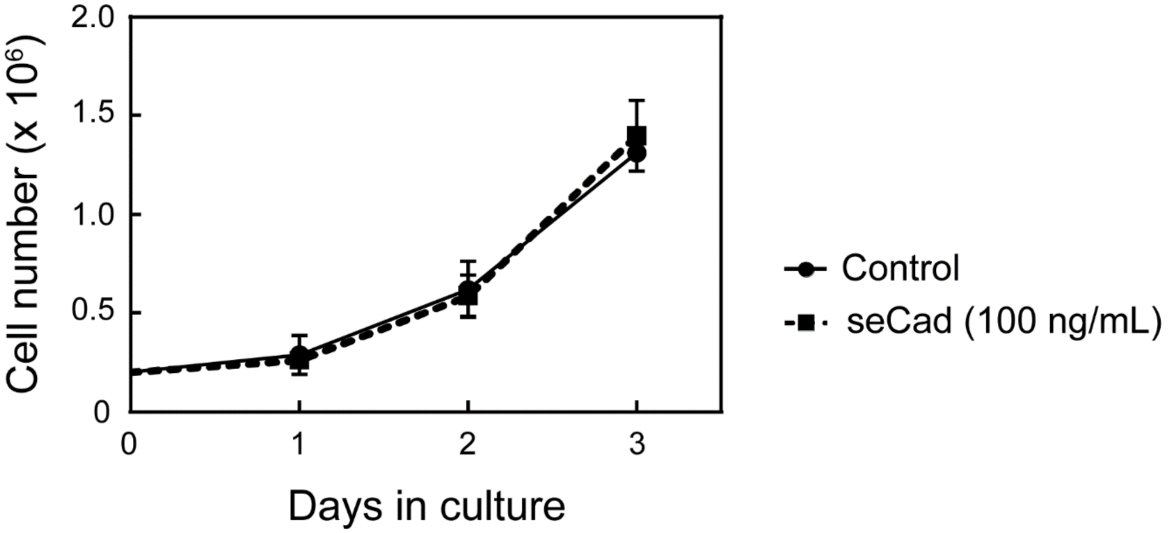

Figure S5. seCad did not affect the proliferation of B16-F10 cells. B16-F10 cells were cultured in the presence or absence of seCad (100 ng/mL). The number of cells was counted every 24 h for the indicated period.

Figure S6, relate to Figure 5

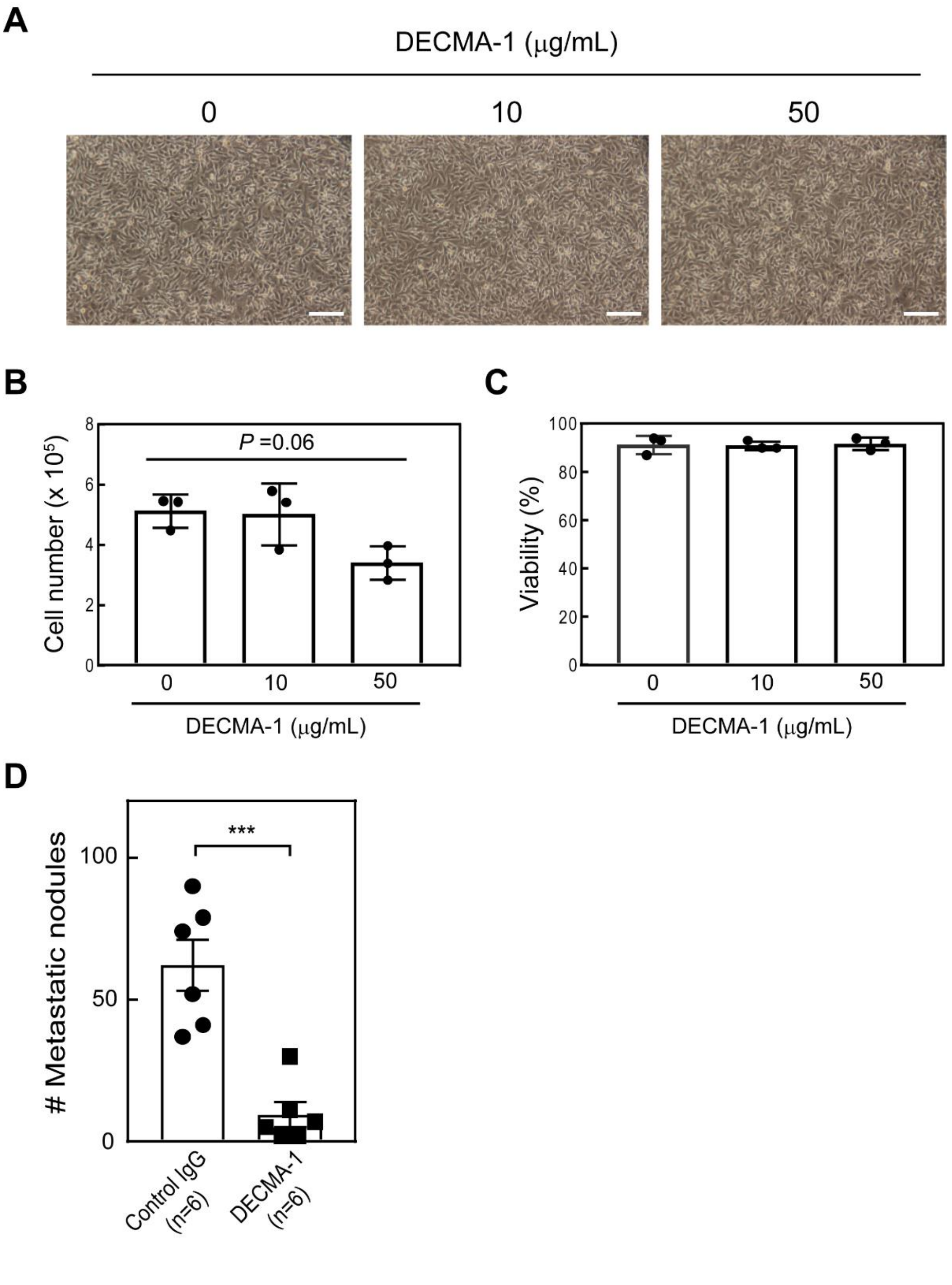

Figure S6. An seCad neutralizing antibody did not affect cell growth or viability. B16-F10 cells were seeded in a 12-well dish (40,000 cells/well). Twenty-four hours later, DECMA-1 was added to the medium at the indicated concentration and cultured for an additional 48 h. (**A**) Representative images of B16-F10 cells. Scale bar, 200 μm. (**B**) The number of cells in each well was counted. (**C**) Cells were stained with propidium iodide (PI), and the proportion of PI-positive cells in each well was measured. Data are presented as the mean values ± SD of triplicate samples. Data were analyzed by one-way ANOVA. Representative results of multiple experiments are shown. (**D**) The number of tumor nodules (total of left and right lobes) was counted. Data are presented as the mean values ± SEM. Data were analyzed by Student's *t*-test. ***$P < 0.001$.

Figure S7, relate to Figure 7

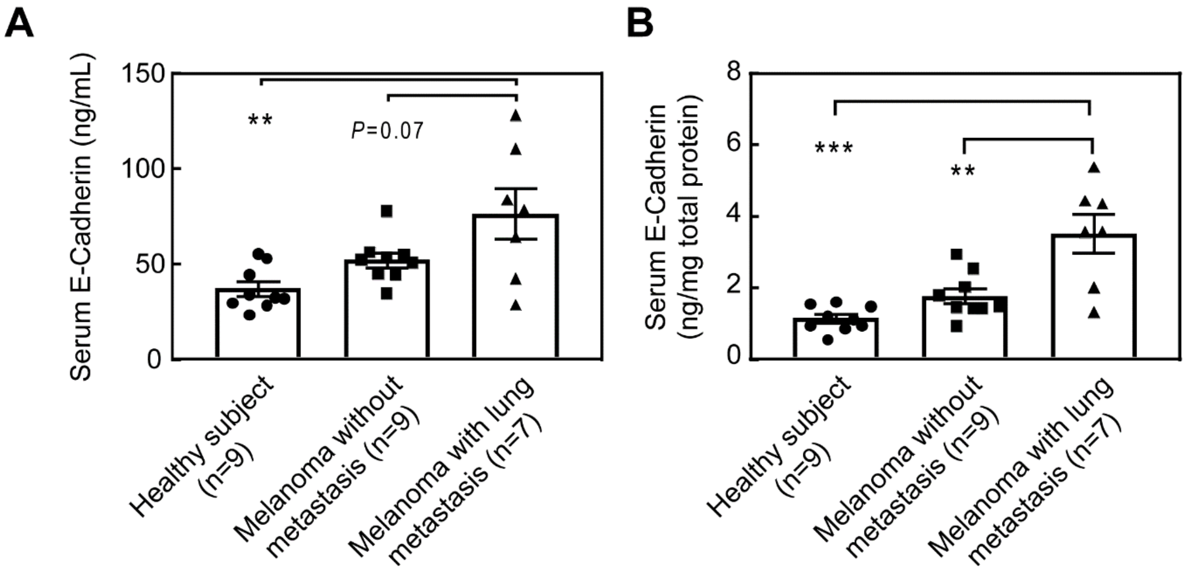

Figure S7. Serum seCad levels in female samples. The serum seCad level in Figure 7 are shown. (**A**) seCad protein per volume. (**B**) The serum seCad levels were normalized to the protein amount in each sample. Data are presented as the mean values ± SEM in each group. Data were analyzed by one-way ANOVA and Tukey post-hoc analysis. $^{**}P < 0.01$ and $^{***}P < 0.001$.
